# Endodontic surgery using 3D-guides in-house manufacturing and trephine burs for osteotomy and root-end resection: a proof-of‐concept clinical study

**DOI:** 10.1007/s00784-025-06660-3

**Published:** 2025-11-26

**Authors:** Nicola Pranno, Gerardo La Monaca, Gianni Di Giorgio, Alessandro Salucci, Maurizio Bossù, Maria Paola Cristalli

**Affiliations:** Department of Oral and Maxillo-Facial Sciences, Sapienza, University of Rome, 6 Caserta St., Rome, 00161 Italy

**Keywords:** 3D printing, Computer-aided design/Computer-aided manufacturing, Guided endodontic surgery, Surgical guide, Apicoectomy

## Abstract

**Objectives:**

The study described a protocol for static-guided endodontic surgery, involving 3D in-house printed guides and trephine burs, to perform osteotomy and root apex resection simultaneously.

**Materials and methods:**

Clinical and radiographic data from 6 patients (9 roots) were evaluated. Cone-Beam Computed Tomography and intraoral scans were imported into the guided surgery software to virtually plan the osteotomy and a 3 mm root-end resection. Based on the virtual plan, surgical guides were designed, and Computer-Aided Design files exported in Standard Tessellation Language format were imported into the slicing software of the 3D printer for manufacturing. Surgeries were carried out under local anaesthesia and involved a mucoperiosteal flap incision and elevation, guided osteotomy and root-end resection using trephine burs, removal of the bone trapdoor, root apex, and periapical lesion with curettes, ultrasonic retropreparation and filling with Mineral Trioxide Aggregate, followed by suturing.

**Results:**

For osteotomy, the size ranged from 20.00 to 93.00 mm² (59.17 ± 32.28 mm²), and the surgical time from 40 to 102 min (73.67 ± 23.00 min). These parameters were influenced by the size of the periapical lesion and the limited accessibility and visibility of the area. No complications occurred, and postoperative pain intensity was mild to moderate. Four patients achieved complete healing at the 6-month follow-up, and two by 12 months.

**Conclusions:**

The current findings supported the use of 3D in-house printed guides and trephine burs in endodontic surgery.

**Clinical relevance:**

3D in-house printed guides in static-guided endodontic surgery offer a more cost-effective alternative to centralised facilities.

## Introduction

Endodontic surgery is recommended to manage persistent and recurrent apical periodontitis in teeth that have previously undergone root canal treatment when orthograde re-treatment is not feasible, has an unfavourable prognosis, or is contraindicated. This approach is also advised in all endodontic conditions that render conventional therapy impractical or inadvisable [1, 2].

Over the years, surgical endodontics has undergone significant improvements due to technological innovations, including illumination and visual magnification devices (loupes, operating microscopes, and endoscopes), Cone-Beam Computed Tomography (CBCT), micro-instruments, ultrasonic tips, and biocompatible root-end filling materials [1–3]. These innovations have enabled more accurate localization of the root apex while reducing harm to surrounding anatomical structures.

Another significant improvement has been the introduction of digital-guided surgery, which combines CBCT imaging with 3D intraoral scanning in guided surgery software to create a digital representation of the patient’s anatomy, facilitating osteotomy and root-end resection planning [4]. Two approaches are available: static and dynamic [5, 6]. In dynamic guided surgery, an optical positioning device drives the drilling procedures through a dedicated computer interface. The integration of surgical handpieces with radiological images allows for real-time visualization of the relationship between the drill and the surgical field, providing the operator with valuable feedback [7]. However, this dynamic approach is not widely adopted due to the high cost of equipment and the need for excellent hand-eye coordination and surgical expertise, as the surgeon, while managing the screen and handpiece during the procedure, is entirely dependent on the computer [8]. Static-guide surgery requires a surgical template designed through virtual planning and produced by importing Computer-Aided Design (CAD) files, exported in Standard Tessellation Language (STL) format, into the slicing software of a 3D printer. Additionally, advancements in 3D printing technology and the commercialization of inexpensive 3D printers have enabled the in-house production of guides, reducing the need for centralized manufacturing facilities, which results in lower costs and better availability.

Static guided surgery provides significant advantages over traditional methods in terms of accuracy, predictability, and healing outcomes, as evidenced by a systematic review of clinical trials involving human subjects [9]. Additionally, a systematic review and meta-analysis of in vitro studies have shown that the precise localization of the root apex had a success rate of 96.8% (CI: 93.0–100%), which was 27 times higher than that of free-hand procedures [10].

More recently, the combination of trephine burs with guided static surgery has enabled simultaneous osteotomy and root-end resection, preventing drilling deviations caused by the cylindrical shape of the bur [11, 12]. This approach is becoming more widespread due to the conservative size of osteotomy and the accuracy of root-end resection, which positively impact operating time, the risk of damaging surrounding structures, postoperative discomfort, and periapical healing of bone defects [13].

The study aimed to describe a novel protocol for static-guided endodontic surgery. In this approach, osteotomy and root-end resection were carried out in a single step using 3D polymeric guides printed in-house and trephine burs.

## Materials and methods

### Study design

This proof-of-concept clinical study retrospectively assessed clinical and radiographic data from six patients with persistent or recurrent apical periodontitis who underwent endodontic surgery, where osteotomy and root-end resection were performed using 3D polymer-based guides printed in-house and trephine burs. Patient data were anonymized. The study protocol was performed in line with the 1964 Declaration of Helsinki on medical protocols and ethics and its later amendments. It was approved by the Department of Oral and Maxillofacial Sciences Board at “Sapienza” University of Rome, Italy (N. 119/2024 - Prot. N. 0001955 del 23.10.2024). The manuscript was drafted following the Strengthening the Reporting of Observational Studies in Epidemiology (STROBE) guidelines.

### Study population

The six patients were treated at the Department of Oral and Maxillofacial Sciences, “Sapienza” University of Rome, Italy, between January 2022 and December 2023. To be included in the study, patients had to meet the following criteria: (1) aged 18 years or older, (2) one tooth treated with guided endodontic surgery and a trephine bur, (3) follow-up of at least 1 year, and (4) adherence to all phases of the treatment and study protocol; (5) availability of clinical files, radiographs, and intraoral photographs from the start of treatment to the end of follow-up. Patients were excluded if their information was incomplete or unavailable. Each patient received detailed descriptions of the treatment and study protocol, including follow-up. Written informed consent was obtained for both the proposed treatment and the publication of data and images for educational and scientific purposes. 

### Digital planning protocol

The diagnostic process involved CBCT to evaluate the characteristics of periapical lesions and adjacent structures, along with intraoral scanning to create a digital model of the dental arch (Medit i700 - Medit Corp., Ltd. - South Korea).

CBCT scans were acquired with a MyRay Hyperion X9 Pro (Cefla S.C., Imola, Italy). The settings were 16 × 13 mm in width and depth, 90 kVp, 4–6 mAs, scan time 6–10 s, resolution at 0.20 voxels, and field of view (FOV) varying according to the region scanned. A high-frequency (“bone”) reconstruction kernel was used. Metal-artifact reduction (MAR) was enabled when metallic restorations were present and disabled when absent.

**Fig. 1:**
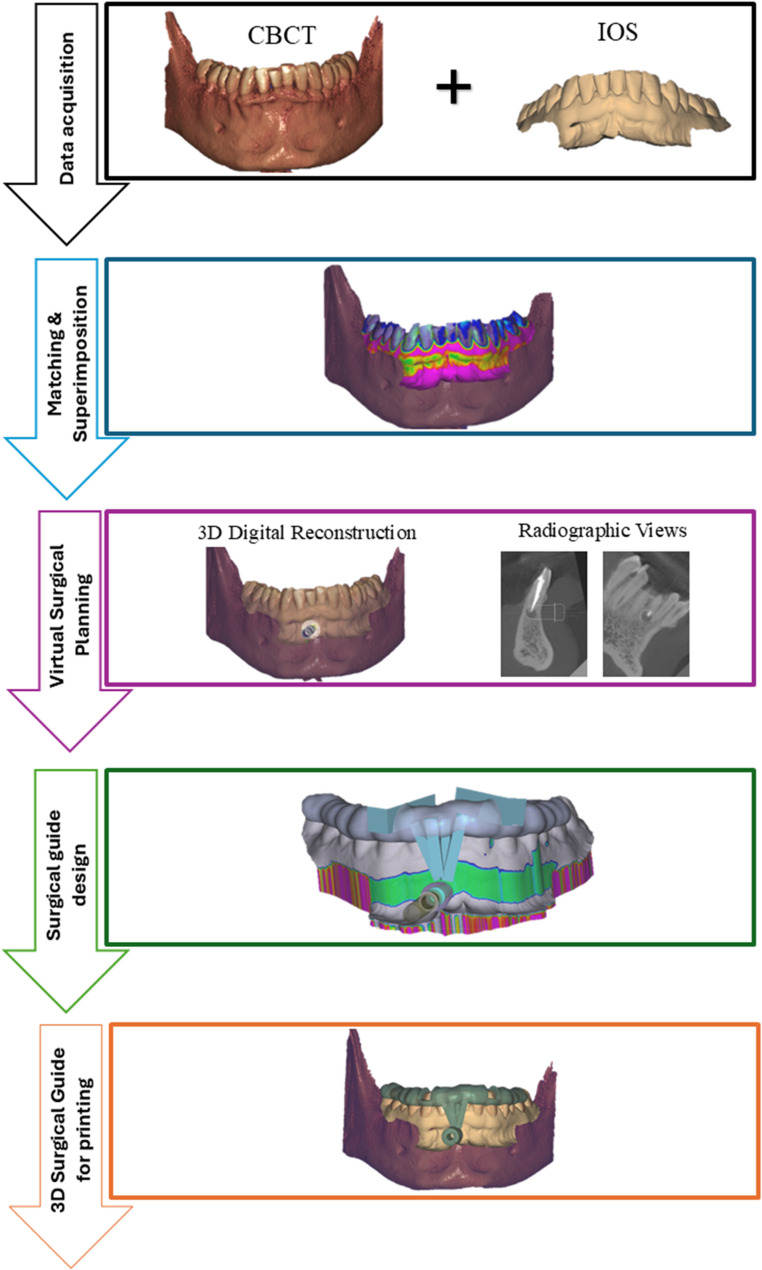
Flowchart of the digital planning protocol

The images, saved as Digital Imaging and Communication (DICOM) files, and intraoral scans in STL format, were imported into the implant planning software (Exoplan 3.1 Rijeka - Exocad GmbH - Germany) to virtually plan the site, size, angulation, and depth of the osteotomy, targeting a 3 mm root-end resection with a zero-degree bevel. Building on the virtual planning, surgical guides were designed. They included dental support to maximize intraoperative stability, observation windows on the occlusal surface for intraoperative fit assessment, and non-metallic sleeves to direct the trephine-bur pathway. Digital analogue implants were utilized to create cylindrical sleeves with an internal diameter matching trephine bur, selected according to the dimensions of the periapical lesion, root-end width, adjacent teeth, and neighbouring anatomical structures. The cylinders were aligned as perpendicular as possible to the tooth’s axis (Fig. 1).

For guide manufacturing [14], the CAD files were exported in STL format and then imported into the slicing software of the Light Crystal Display (LCD) 3D printer (AccuFab-L4D - Shining 3D Tech. Co., Ltd., Hangzhou, China), set up with at 45° orientation, 50 μm layer printing and 405 nm wavelength. Surgical guides were printed using a transparent, biocompatible photopolymer resin (Shining 3D Surgical Guide Resin SG01), specifically designed for 3D printing of dental models, which are non-implantable and suitable for use with body contact for up to 24 h. After 3D printing, guides underwent post-processing using the Shining 3D FabWash system (Shining 3D Tech. Co., Ltd., Hangzhou, China) to remove any uncured resin from the surface, and the Shining 3D FabCure 2 Post-curing unit (Shining 3D Tech. Co., Ltd., Hangzhou, China) to enhance the material’s mechanical properties. The washing was an automatic process involving a 6-minute bath in 90% isopropyl alcohol (IPA) and air drying overnight. For post-curing, each side was exposed to a powerful UV light emitted by 30 multi-directional LEDs for 5 min. Following post-processing, guides were sealed in sterilization pouches, autoclaved at 134 °C and 2 bar for 10 min, and stored at room temperature.

### Surgical protocol

**Fig. 2:**
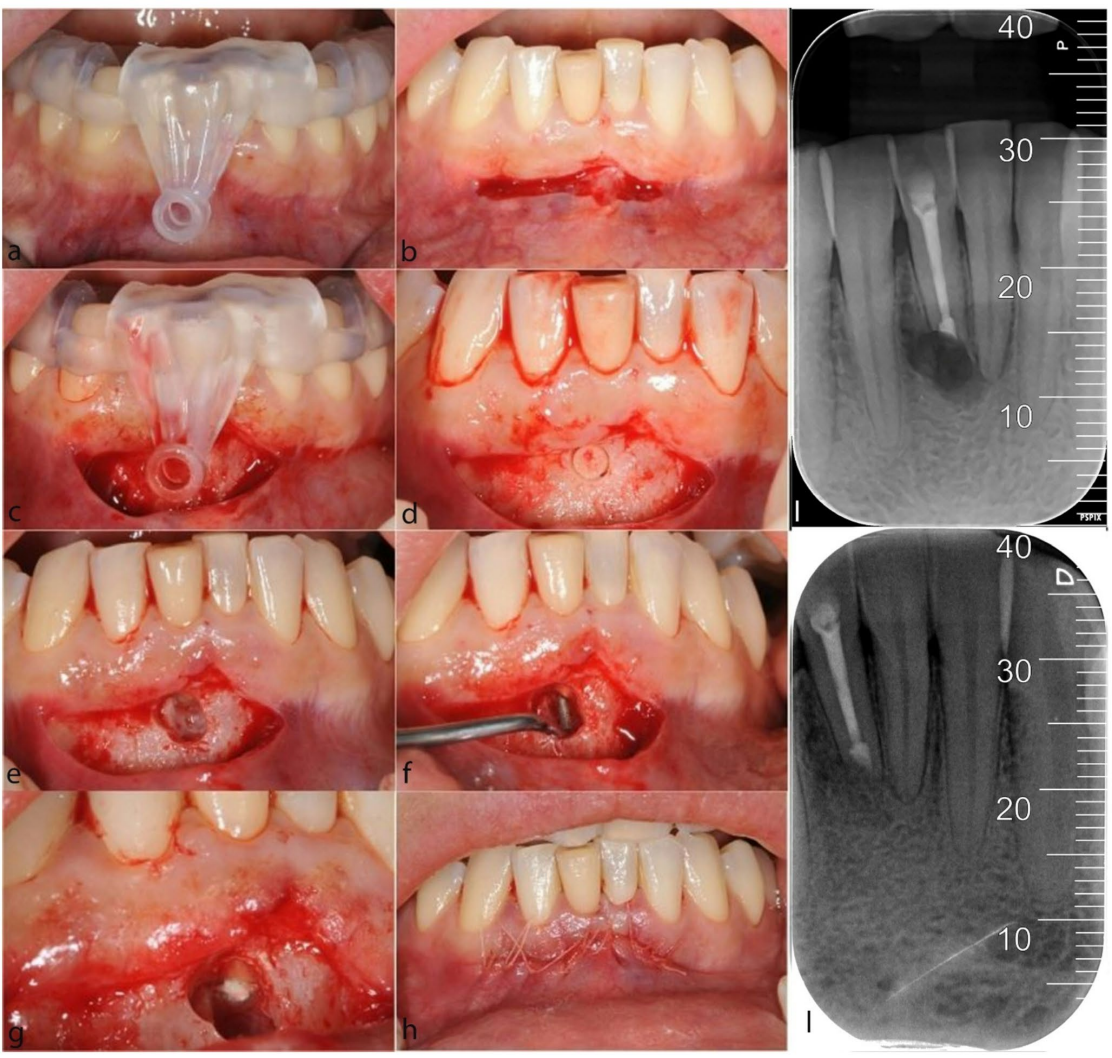
Surgical procedure: **a** surgical guide fitting; **b** incision of linear paramarginal mucoperiosteal flap; **c** surgical guide in place to guide the trephine bur; **d** the bone trapdoor; **e** bone cavity after removal of trapdoor, root apex and periapical lesion); **f** retropreparation; **g** retrofilling; **h** suture; **i** postoperative periapical radiograph; **l** periapical radiograph at 6-month follow-up showing bone healing

All surgical procedures were performed by the same surgeon (G.L.M.), experienced in guided endodontic surgery. Before starting the surgical procedure, the guide was placed inside the patient’s mouth to verify proper fit and the absence of tilting or micro-movements (Fig. 2a). Subsequently, under high magnification (Opmi Pico, Zeiss, Germany), local anaesthesia was administered using Ultracain D-S Forte 1:100000 (Sanofi-Aventis GmbH), and a mucoperiosteal flap was incised. The size and shape of the flap were determined by surgical accessibility, prosthetic restorations, and the width of the keratinized gingiva (Fig. 2b). After raising the full-thickness flap, the surgical template was positioned (Fig. 2c), and the trephine bur (Hager & Meisinger, Neuss, Germany) was inserted into the guiding sleeve to perform the osteotomy and apical resection. The trephine bur was operated at 600 rpm and 35 Nm of torque, employing an inside-out drilling technique under continuous external irrigation with a cooled saline solution. The bone trapdoor (Fig. 2d), root apex, and periapical lesion were removed with a curette, and, if necessary to allow instrumentation access to the resected root, the osteotomy was widened using tungsten carbide fissure burs mounted on a low-speed handpiece under irrigation (Fig. 2e). The retrograde preparation was carried out using ultrasonic tips (SONICflex 2008; KaVo Dental GmbH, Biberach, Germany) (Fig. 2f). Haemostasis at the surgical site was achieved using cotton pellets soaked in anaesthetic with adrenaline 1:100000, and the retrograde filling was performed with Mineral Trioxide Aggregate (MTA - Dentsply Tulsa Dental Specialities, Johnson City, TN) (Fig. 2g). Before repositioning and suturing the flap with 5.0 monofilament (Ethicon, USA) (Fig. 2h), a periapical radiograph was taken to check for the absence of foreign material and proper apical sealing (Fig. 2i). The sutures were removed after two weeks. The antibiotic protocol involved administering 875 mg of amoxicillin and 125 mg of clavulanic acid (Augmentin, GlaxoSmithKline, London, UK) twice daily for five days, starting 1 h before surgery. Perioperative antibiotic prophylaxis was administered to prevent surgical site infections and was justified by the clean-contaminated procedure involving an infected area at risk of bacteraemia, as well as mucosal disruption, exposure of the jawbone, and opening of marrow spaces over an extended period [15]. Pain relief was provided with 200 mg of ketoprofen (Ibifen, I.B.I. Aprilia, Latina, Italy) up to three times daily as needed. Postoperative oral care instructions included rinsing with chlorhexidine digluconate 0.2% (Curasept, Curaden Healthcare srl, Saronno, Varese, Italy) mouthwash twice daily and gentle tooth brushing until the sutures were removed.

### Clinical and radiographic assessment

The recorded data included age, gender, tooth type, number of treated roots, periapical lesion area, osteotomy size, timing of surgery, postoperative pain and complications, as well as clinical and radiographic healing outcomes. The size of preoperative periapical lesions and osteotomies, given their irregular shape, was calculated by multiplying the maximum mesiodistal (width) and apicocoronal (height) dimensions, with the results expressed in mm² [16]. Measurements for periapical lesions were obtained on CBCT coronal multiplanar reconstructions (fixed window/level), independently by two trained investigators not involved in patient treatment. The investigators’ calibration was performed before the study using 50 CBCT scans of periapical lesions, and the Intraclass Correlation Coefficient (ICC) was used to evaluate inter- and intra-operator variability. When the ICC indicated good reliability (ICC > 0.75), the investigators were considered calibrated and began the radiograph evaluations. Discrepancies were resolved through discussion, and when full agreement was not reached, for differences exceeding 0.1 mm, a third investigator was consulted, and the consensus value was recorded. Osteotomies were measured intraoperatively by the surgeon at the end of the bone removal step, using a periodontal probe (PCP15, Hu-Friedy, Chicago, IL, USA). Surgical time was recorded using an electronic timer by someone not involved in the study, tracking each step from the start of the flap incision to the end of the suture. For multi-rooted teeth, the timing assessment was based on the overall duration required to complete each surgical phase, regardless of the number of treated roots.

Postoperative pain was assessed using the Numerical Rating Scale-11 (NRS-11), which comprises 11 equal intervals (scores 0–10), ranging from 0 (no pain) to 10 (worst pain) [17]. At the end of the intervention, patients received the scale and were instructed to record their pain intensity every hour from 1:00 p.m. (approximately 2 h after surgery) until 11:00 p.m. on the operative day, and from 8:00 a.m. to 11:00 p.m. for the following two days. This method of evaluating postoperative pain intensity, although depending on the patient’s collaboration, was considered valid and reliable for unidimensional self-assessment of pain, which is an inherently subjective experience.

Clinical healing is defined as the absence of signs and symptoms, including pain, swelling, and tenderness upon palpation in the apical area, tooth tenderness to percussion, tooth mobility, periodontal pockets, and the presence of a sinus tract or abscess. Radiographic assessment of healing was performed using periapical radiographs taken with the parallel long-cone technique and a standardized film holder (Rinn Centratore XCP Evolution 2003, Dentsply, Rome, Italy) immediately after surgery and at 6 and 12 months post-operatively. The exposure settings were 70 kV for tube voltage, 7 mA for tube current, 1 s for exposure time, and a focal spot size of 0.5 mm.

Radiographic healing was classified as either complete or incomplete, depending on whether the immediate postsurgical radiolucency was fully or partially reduced. It was considered uncertain or unsatisfactory if the radiolucency remained unchanged or increased in size compared to its initial postsurgical dimensions [18, 19]. The treatment outcome was associated with both clinical and radiographic healing. Success was characterised by the lack of clinical signs and/or symptoms, combined with radiographic evidence of either complete or partial healing. Failure was defined by the presence of clinical signs and/or symptoms or radiographic evidence of uncertain or unsatisfactory healing.

### Statistical analysis

Standard statistical analysis software (version 27.0; IBM SPSS Statistics, IBM Corp., Armonk, NY, USA) was utilised for data analysis. Continuous variables are presented as means with standard deviations (SD), ranges (minimum–maximum), and 95% confidence intervals (CI) (calculated using the t-distribution with df = *n* − 1). Categorical variables — such as gender distribution, postoperative swelling incidence, and radiographic healing outcomes — were summarized as frequencies and percentages. Pain intensity was summarized daily as the patient-level mean NRS, with both mean (SD) and median (IQR) reported. To assess trajectories, an area under the curve (AUC) over 72 h was calculated per patient as: AUC = 10× (Day 1 mean, 13:00–23:00), + 15 × (Day 2 mean, 08:00–23:00), + 15 × (Day 3 mean, 08:00–23:00).

## Results

Six patients (3 females and 3 males) with a total of 9 roots met the inclusion criteria. The mean age of the patients was 53 ± 9.01 years (range, 41–65 years). The demographic data and baseline clinical characteristics, as well as the surgical findings, of the study population are presented in Tables 1 and 2, respectively.

**Table 1:** Demographic data and baseline clinical characteristics of the study population

Patient	Sex	Age years	Tooth	Rooth to treat	Lesion area mm^2^	Cortical thikness mm	Osteotomy size mm^2^
1	F	60	1.1	single	45,00	1,00	42,00
2	M	65	1.7	MB, DB, P	66,00	4,00	80,00
3	F	45	4.1	single	12,00	2,00	20,00
4	M	52	4.7	single	110,00	2,00	90,00
5	M	55	2.6	MB, DB	72,00	1,50	93,00
6	F	41	1.4	single	30,00	4,00	30,00

*F* female, *M *male, *MB *mesial-buccal, *DB *distal-buccal, *P *palatal

**Table 2:** Surgical findings of the study population

Surgical findings	Patient 1	Patient 2	Patient 3	Patient 4	Patient 5	Patient 6
Time for flap incision and dissection	5	5	2	10	4	7
Time for osteotomy	8	10	4	17	6	4
Time for lesion removal	14	28	4	31	10	20
Time for retropreparation	12	27	9	14	9	9
Time for hemostasis and retrofilling	19	20	10	14	23	16
Time for suture	13	12	11	15	13	7
Total time	71	102	40	101	65	63
Mean pain at day1	3	3	3	5	4	4
Mean pain at day 2	2.50	2	2	3.50	3	3
Mean pain at day3	2.50	1	1.50	4	2	3
Postoperative complications	0	0	0	swelling	swelling	0
Time to complete healing	6	6	6	12	12	6

Surgical timings were given in minutes; postoperative pain was indicated as a score on a 0–10 scale; time to complete healing was expressed in months

**Fig. 3:**
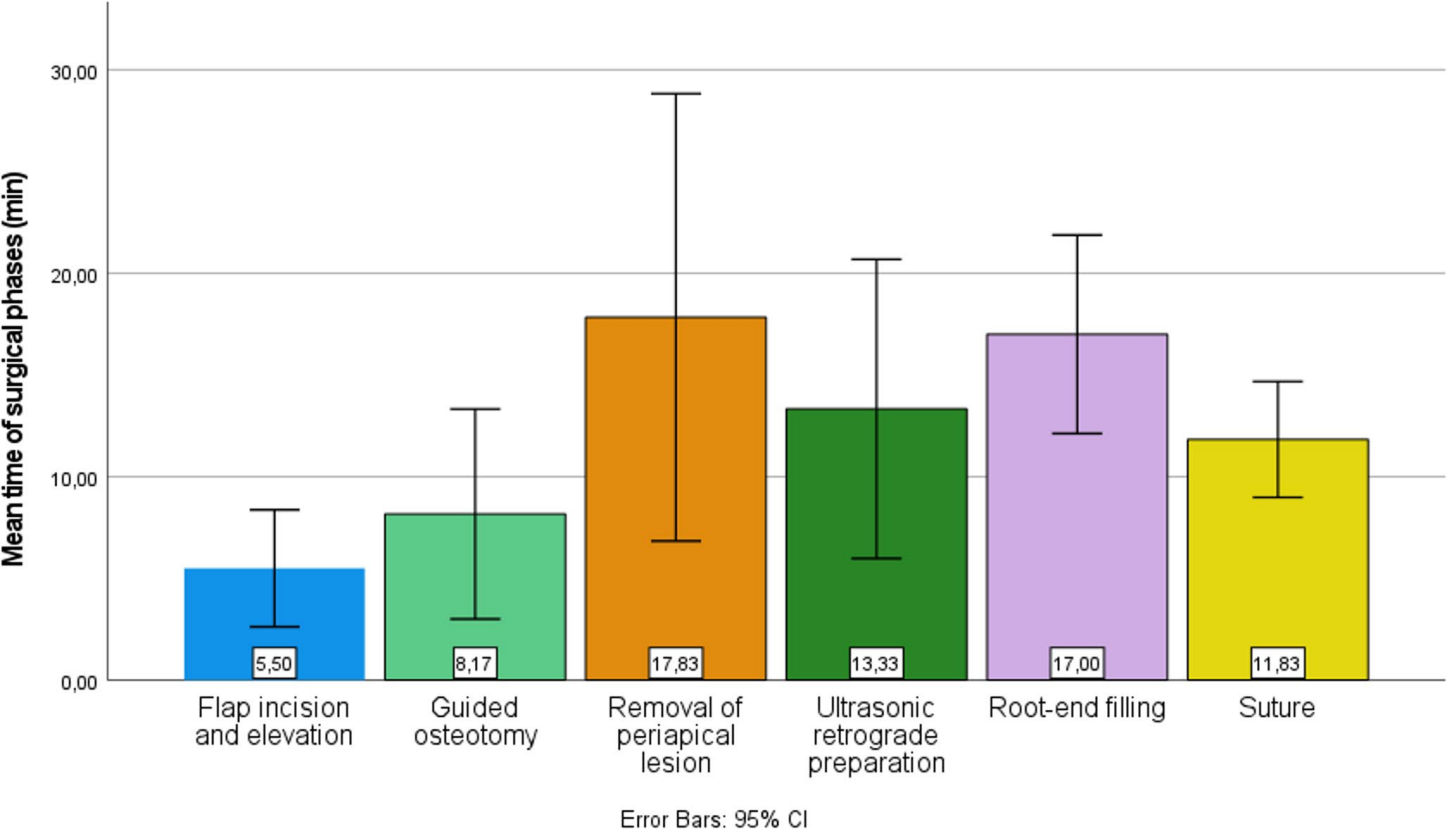
The bar graph illustrates the mean duration (in minutes) of each surgical phase

The area of periapical lesions ranged from 12.00 to 110.00 mm², with a mean of 55.83 ± 34.65 mm² (95% CI: 19.47–92.20 mm²). The mean size of the osteotomy was 59.17 ± 32.28 mm² (95% CI: 25.29–93.04 mm²; range: 20.00–93.00 mm²), and it was affected by the space required to perform the retrograde preparation and filling of the resected root. The osteotomy and apical resection were performed in a single stage for all six procedures, showing consistency between the actual and planned root apex positions. The timing of surgical procedures ranged from 40 to 102 min with a mean total operative time of 73.67 ± 24.00 min (95% CI: 48.48–98.85 min). The significant variation as influenced by the size of the periapical lesions and the presence of thicker cortical bone, as well as site-specific factors such as limited accessibility and poor visibility (Fig. 3). The most extended duration was recorded for molars, mainly due to the anatomical complexities of their multiple roots and canal morphology [20]. Among the individual stages, the shortest was the flap incision and elevation (mean 5.50 ± 2.74 min; 95% CI: 2.63–8.37 min), followed by the guided osteotomy with simultaneous root-end resection (mean 8.17 ± 4.92 min; 95% CI: 3.01–13.33 min). The longest stage was the removal of the periapical lesion (mean 17.83 ± 10.48 min; 95% CI: 6.84–28.83 min), followed by ultrasonic retrograde preparation (mean 13.33 ± 7.00 min; 95% CI: 5.98–20.68 min) and root-end filling (mean 17.00 ± 4.65 min; 95% CI: 12.12–21.88 min). The time required for suturing showed little variation, ranging from 7 to 15 min (mean 11.83 ± 2.71 min; 95% CI: 8.98–14.68 min).

Pain diaries were completed for all participants (6/6). Patients experienced mild to moderate postoperative pain, with scores ranging from 1 to 5 on the NRS-11. Pain levels gradually declined from the first day (mean 3.25 ± 0.76; median 3.5; IQR 3.0–4.0) to the second day (mean 2.67 ± 0.61; median 2.75; IQR 2.25–3.0), and then to the third day (mean 2.33 ± 1.08; median 2.25; IQR 1.75–2.75). To summarize the trajectories, the median three-day area under the curve (AUC) was 110.0 NRS·h (IQR 93.75–122.5), with a mean of 111.7 ± 32.2 NRS·h. This reflected a mean pain intensity of approximately 2.75/10 over the 40 hours monitored, suggesting a mild overall pain burden. Postoperative recovery was uneventful, except for two cases involving molars, where swelling was associated with the size of the periapical lesions and the extent of the osteotomy. Complete radiographic and clinical healing was achieved in all six patients, with four cases at the 6-month follow-up and two by 12 months. 

## Discussion

 The available literature on digital guides in endodontic surgery offers limited and low-quality evidence. Most studies are in vitro or ex vivo simulations, case reports, and case series, with notable differences in purpose, methodology, procedures, and follow-up [21]. In this context, the present proof-of-concept study aimed to provide initial indications of the effectiveness of a novel protocol for static-guided endodontic surgery. This protocol involved the utilisation of in-house 3D-printed guides and trephine burs to carry out osteotomy and root apex resection in a single step In many studies, the use of surgical guides demonstrated improved accuracy in reaching the root apex during osteotomy when compared to three-dimensional presurgical visualization alone. Pinsky et al. identified a statistically significant difference in the apical distances of the osteotomies from the intended objectives in their preclinical study using a dry mandible model [22]. The recorded mean discrepancy was 0.79 ± 0.33 mm for the guided apicoectomy and 2.27 ± 1.46 mm for the freehand drilling. Similarly, the accurate localization and resection of the root end, perpendicular to the longitudinal axis, during guided surgical endodontic procedures, were demonstrated by a split-mouth cadaver study [23] In a series of 14 root-end resections performed on 11 patients, the combination of trephine burs and 3D-printed guides for performing single-step osteotomy and root-end resection resulted in efficient, targeted osteotomies with predictable site, angulation, and depth of preparation [24]. An in vitro comparison between the trephine bur and pilot drill regarding their efficiency for osteotomies and apicoectomies revealed comparable performance, with recorded distal endpoint deviations of 1.53 ± 0.51 mm for the pilot drill and 1.31 ± 0.46 mm for the trephine bur [25]. Furthermore, it was reported that the higher precision of single-step osteotomy and root-end resection in the guided approach, compared to traditional endodontic microsurgery, resulted in a significantly lower volume of over-resection and root removal. This minimally invasive access led to shorter surgery times, less postoperative discomfort for patients, and more favourable healing outcomes [26, 27]. The mean surgical time for osteotomy and root-end resection, as documented in a randomised controlled trial involving 60 patients, was 121.75 ± 130.24 s and was also shorter in the static guided surgery group compared to the dynamic guided surgery group [28] The intensity of postoperative pain and its gradual decline observed in the study population aligned with the findings of a retrospective analysis involving 173 patients, which aimed to determine the prevalence and characteristics of postoperative pain following endodontic microsurgery and identify potential predictors of severe pain [29]. In this analysis, most patients experienced mild or moderate pain during the first two days after surgery, with a gradual decrease thereafter. Additionally, no correlation was found between severe pain and the anterior or posterior position of teeth, lesion size, or the presence of fenestration. Conversely, sex, age, and bone thickness emerged as significant predictors. The postoperative pain scores were consistent with those reported in a randomized clinical trial evaluating the impact of piezosurgery and trephine burs on postoperative pain during guided endodontic microsurgeries of mandibular first molars. All 20 patients experienced similar levels of pain, regardless of the instruments used, indicating that the trephine’s low speed and the irrigation during the osteotomy procedure produced effects comparable to the less traumatic piezosurgery [30] However, achieving success depends on the accuracy of surgical guides, which is crucial and relies on the diagnostic and manufacturing processes adopted [31]. In the protocol applied in the present study, 3D reconstructions of patient jaws were created by combining high-resolution CBCT imaging with high-accuracy intraoral scans. This method facilitated the assessment of the size and location of periapical lesions, the thickness of adjacent bone tissue, and the proximity to anatomical structures, including the mental nerve, sinus cavity, and teeth. The gathered information was utilised for diagnosis, planning interventions, and designing surgical templates. The guide’s design incorporated dental support to ensure intraoperative stability, observation windows on the occlusal surfaces of the teeth to control the fit during the operation, and sleeves for trephine burs to manage the drilling depth, length, and angle for root-end resection, as well as fenestrations for heat dissipation [32]. The innovation involved the in-house production of 3D-printed, polymer-based guides. This printing method simplifies procedures and reduces costs compared to central manufacturing facilities, as the devices routinely used in implant surgeries are readily available in dental practices Similar to other investigations, the present study evaluated treatment outcomes one year after surgery, focusing on clinical signs and symptoms, as well as the assessment of radiolucency in periapical radiographs. Most papers considered a one-year follow-up using conventional periapical radiography adequate for identifying the success or failure of healing [33]. The decision to use conventional radiographs was guided by the risk-benefit ratio of radiation exposure, which contraindicated the use of CBCT imaging for routine follow-ups after endodontic procedures, except in more complex cases with slower healing or when assessing the healing stage was difficult to determine [34, 35]. A systematic review comparing the healing assessment of endodontic surgery using periapical radiographs and CBCT concluded that the diagnostic efficacy of CBCT remains uncertain. Many studies have found insignificant differences between the two techniques, while others have reported that 3D radiography provides a more comprehensive evaluation of healing [36]. Furthermore, the diagnostic validity of periapical radiographs versus CBCT was demonstrated by histopathological examinations of 74 teeth subjected to endodontic reoperation due to a diagnosis of unsuccessful healing at a 7-year follow-up [37]. In the study, histopathological examination of the periapical soft tissues revealed the presence of scar tissue, with no evidence of periapical inflammation, in more than 40% of cases. When comparing the histopathological findings, periapical radiographs achieved an accurate diagnosis in 63% of cases, whereas CBCT correctly identified it in 58% of cases. The authors concluded that caution should be exercised when using CBCT to assess periapical healing following endodontic surgery and during follow-ups, and that patient symptoms should be regarded as a reliable indicator of periapical inflammatory lesions. The correlation observed between the healing timing, periapical lesion dimensions, and osteotomy size was consistent with data from a prospective observational cohort study evaluating apical surgery outcomes over 4 to 8 years [38]. In the investigation, the only variable that significantly affected bone healing was the presence of preoperative radiolucency measuring less than 5 mm. Conversely, a radiolucency exceeding 5 mm was linked to a greater likelihood of fibrous tissue formation and delayed healing. More recently, Kim et al. reported a higher success rate in cases with lesions smaller than 6mm in diameter in each of the three dimensions. However, the differences were not statistically significant [39] Considering the literature reports mentioned above, endodontic surgery performed with a guided template and a trephine bur offers several advantages over the conventional approach. These include: more accurate localisation of the root apex, resulting in minimally invasive preparation; a significant reduction in the volume of bone preparation; minimisation of intra- and postoperative complications, such as bleeding or damage to adjacent teeth and anatomical structures; shortened surgical time; more favourable postoperative healing; and a reduced risk of infection, leading to a better prognosis and more predictable results, regardless of the clinician’s experience. However, the approach presents certain disadvantages, such as potential inaccuracies in the surgical guide, difficulties in application within posterior regions due to limited buccal space, lack of real-time visualisation, and a fixed drill position that cannot be modified during the procedure.

## Limitations

This clinical study, which aimed to describe a novel protocol for static-guided endodontic surgery, presented some limitations, mainly due to its proof-of-concept design, small sample size, and the evaluation method using two-dimensional periapical radiographs. Additionally, the accuracy in terms of deviations in the entry point, angle, and depth between digital planning and surgery was not assessed. Nevertheless, in all six procedures, the osteotomy and apical resection were completed in a single step, confirming that the planned position of the root apex matched the actual location. Finally, the perioperative antibiotic prophylaxis outlined in the protocol should not be regarded as mandatory, as it was based on personal experience and considerations due to the lack of explicit guidelines on antibiotic use to prevent surgical site infections.

## Conclusion

Despite the limitations, the current preliminary findings supported the combined use of 3D-printed in-house polymer-based guides and trephine burs in endodontic surgery. However, further validation of this approach through large-scale, controlled clinical trials with long-term follow-up is necessary.

## Data Availability

No datasets were generated or analysed during the current study.
